# Bairui granules versus Reyanning granules in adults with acute bronchitis: a multicenter, randomized, double-blind, double-dummy, comparative trial

**DOI:** 10.3389/fphar.2026.1860478

**Published:** 2026-07-16

**Authors:** Youqiang Wu, Guantong Shen, Shuyang Ji, Guoxing Liu, Weicheng Nie, Bei Xue, Chen Zuo, Yingjie Du, Jingyi Qi, Mingzhe Wang, Chengjun Ban, Miao Cheng

**Affiliations:** 1 Respiratory Department, Dongzhimen Hospital, Beijing University of Chinese Medicine, Beijing, China; 2 Respiratory Department, Beijing University of Chinese Medicine Third Affiliated Hospital, Beijing, China; 3 OISE Department of Applied Psychology & Human Development, University of Toronto, Toronto, ON, Canada

**Keywords:** acute bronchitis, Bairui granules, randomized controlled trial, traditional Chinese medicine, wind-heat invading lung syndrome

## Abstract

**Background:**

Bairui granules, a monotherapy derived from *Thesium chinense* Turcz., are widely used in respiratory tract infections, yet high-quality clinical evidence for their effectiveness in acute bronchitis is lacking.

**Purpose:**

To evaluate the effectiveness and safety of Bairui granules in patients with acute bronchitis (Wind-heat invading lung syndrome).

**Study Design:**

The study (ChiCTR2200063906) was a multicenter, randomized, double-blind, double-dummy, comparative trial.

**Methods:**

Eligible participants from seven hospitals were randomized 2:1 to receive either Bairui granules plus placebo matching Reyanning Granules (experimental group) or Reyanning granules plus placebo matching Bairui Granules (control group) orally for 7 days. The primary endpoint was cough resolution rate after treatment. Secondary endpoints included time to cough and sputum resolution, area under the curve (AUC) for cough and sputum severity, bronchitis severity score (BSS), and traditional Chinese medicine (TCM) symptom scores.

**Results:**

Of 162 randomized patients, 161 completed the trial (107 experimental, 54 control). The cough resolution rate was higher in the experimental group than in the control group (72.22% vs. 35.19%; *P* < 0.0001). The time to cough resolution was 144 h (120,156) versus 168 h (138, 168) (*P* = 0.001). AUC for cough severity was 184.3 versus 214.3 score·h (*P* = 0.023). The BSS improvement at Day 9 was −4.82 (3.54) versus −4.30 (0.71) (*P* = 0.068). The daytime cough TCM score improved by −6 (−6, −6) versus −6 (−6, −3) (*P* = 0.003), and the sputum score improved by −3 (−6, −3) versus −3 (−3, −3) (*P* = 0.031). Greater improvements were also observed in aversion to wind symptoms (Day 4) and sore throat symptoms (Day 9) in the experimental group. Adverse-event rates were similar between the two groups. All events were mild to moderate in severity and reversible.

**Conclusion:**

Bairui granules resulted in a higher cough-resolution rate and faster cough resolution than Reyanning granules in adults with acute bronchitis. The treatment was well tolerated.

**Clinical Trial Registration:**

https://www.chictr.org.cn/hvshowproject.html?id=193073&v=1.0, identifier ChiCTR2200063906.

## 1 Introduction

Acute bronchitis is a common clinical condition, accounting for approximately 10% of outpatient healthcare visits annually (Mulhem et al., 2025; Harris et al., 2016). Although typically self-limiting, cough and sputum symptoms often persist for 1–3 weeks, which frequently causes sleep disturbance, time off work, and repeated medical visits (Kinkade and Long, 2016). Antibiotics are frequently prescribed despite predominantly viral etiology and limited benefit, contributing to antimicrobial resistance and avoidable adverse effects (Turner et al., 2025). In addition, inadequate or delayed management in susceptible individuals may result in disease exacerbation, respiratory failure, septic shock, even death (Ngu et al., 2019). These issues highlight the need for effective, well-tolerated non-antibiotic therapies that can shorten symptom duration and reduce disease burden in acute bronchitis.

In traditional Chinese medicine (TCM) theory, acute bronchitis corresponds mainly to patterns such as “wind-heat invading lung syndrome”, characterized by cough with yellow sputum, sore throat, thirst, nasal congestion (rhinobyon), and aversion to wind (Wang et al., 2013). Bairui granules are a single-botanical drug preparation derived from *Thesium chinense* Turcz. They are widely used in clinical practice for the treatment of acute bronchitis, pneumonia, rhinitis, and pharyngitis (Chai et al., 2024; Li et al., 2021). UPLC analyses show that Bairui granules contain alkaloids and organic acids with antibacterial activity, and kaempferol-rich flavonoids that provide anti-inflammatory and immunomodulatory effects—together helping reduce airway inflammation, suppress infection (Li et al., 2021; Zhang et al., 2024; Peng et al., 2024). However, evidence regarding the effects of Bairui granules on acute bronchitis in the real-world settings remain limited. Reyanning Granules are a multi-botanical drug preparation listed in the *Chinese Pharmacopoeia* (2020 edition) and are commonly used in China for respiratory conditions characterized by wind-heat manifestations. In previous clinical studies, Reyanning preparations have been used for respiratory infections and wind-heat syndrome-related conditions, providing a clinical basis for their use as an active comparator (Xu et al., 2023a; Liu et al., 2023). Therefore, we conducted a multicenter, randomized, double-blind, double-dummy, comparative trial to further investigate the effects of Bairui granules versus Reyanning granules in patients with acute bronchitis.

## 2 Methods and materials

Our study followed the reporting guidelines of CONSORT 2025 (Hopewell et al., 2025) and ConPhyMP (Heinrich et al., 2022).

### 2.1 Study design

This multicenter, randomized, double-blind, double-dummy, comparative trial was conducted at seven sites in China (Supplementary Table S1) to evaluate the effectiveness and safety of Bairui granules in the treatment of acute bronchitis. The study was registered in the Chinese Clinical Trial Registry (ChiCTR2200063906; registration date: 20 September 2022) and approved by the Ethics Committee of Dongzhimen Hospital, Beijing University of Chinese Medicine (approval No. 2021DZMEC-212-02). The final trial protocol (version [P2021-09-BDY-11-V02], dated [18 February 2022]) were finalized before enrollment of the first participant. All procedures were performed in accordance with the principles of the Declaration of Helsinki. Written informed consent was obtained from all participants prior to enrollment.

### 2.2 Participants

Patients with acute bronchitis were eligible if the diagnosis was established according to the *Chinese Medical Association Guidelines for the Primary Diagnosis and Treatment of Acute Tracheobronchitis (2018)* and if they met the TCM diagnostic criteria for the wind-heat invading the lung syndrome according to the *Diagnosis and treatment guideline for Chinese medicine on acute tracheobronchitis (2015)*. The required TCM manifestations included cough, sputum characteristics, sore throat, thirst, nasal congestion, aversion to wind, and related wind-heat symptoms, as specified in the diagnostic guideline. Before patient enrollment, investigators from all participating centers received unified protocol training on the TCM pattern criteria. Patients also had to be 18–65 years of age, present within 3 days after symptom onset, and have a daytime or nocturnal cough symptom score of at least 2.

Key exclusion criteria were an axillary temperature of 38.5 °C or higher; suspected or confirmed pneumonia or other clinically relevant respiratory diseases associated with cough (e.g., bronchiectasis, asthma [including cough-variant asthma], lung cancer, or acute exacerbation of chronic obstructive pulmonary disease); laboratory findings suggestive of bacterial infection (total white-cell count >11 × 10^9^/L or neutrophil percentage >80%); recent use of antibiotics or medications or therapies likely to affect cough symptoms before the baseline visit; clinically significant hepatic or renal dysfunction; pregnancy or breastfeeding; participation in another clinical trial within 30 days before screening; or any condition that, in the judgment of the investigators, could interfere with participation, adherence, or follow-up. Full eligibility criteria are provided in the Supplementary Appendix.

### 2.3 Randomization and blinding

Eligible participants were randomly assigned in a 2:1 ratio to receive Bairui Granules or Reyanning Granules. The randomization sequence was generated by an independent statistician using a block randomization design implemented with the PROC PLAN procedure in SAS software (version 9.4). Allocation concealment was maintained using sequentially numbered, sealed, opaque envelopes and corresponding pre-labeled medication kits prepared according to the randomization sequence. After confirmation of eligibility and completion of baseline assessments, the investigator opened the next envelope in numerical order and dispensed the corresponding medication kit. The randomization sequence and treatment codes remained inaccessible to participants, investigators, outcome assessors, and trial statisticians involved in blinded analyses until database lock and formal unblinding.

### 2.4 Material

Bairui granules (batch number: S220401; Anhui Jiuhua Huayuan Group Pharmaceutical Co., Ltd., China) are a single-botanical drug preparation made from the dried whole plant of *T. chinense* Turcz. [Santalaceae; Thesii Herba]. The scientific and medicinal names of the source plant material were verified against the Medicinal Plant Names Services database (MPNS; http://mpns.kew.org). Previous phytochemical investigations using UPLC–MS/MS have identified a broad range of plant metabolites in Bairui Granules, including alkaloids, flavonoids, organic acids, glycosides, terpenes, and lactones, with flavonoids, particularly kaempferol, reported as major marker metabolites (Li et al., 2021; Li et al., 2024; Zhang et al., 2024; Peng et al., 2024). The study batch complied with the National Medical Products Administration standard YBZ10642009, and its chemical fingerprint was confirmed by targeted UPLC analysis (Supplementary Figure S1).

Reyanning granules (batch number: 2107502; Hunan Zhengqing Pharmaceutical Group Co., Ltd., China), used as the comparator in this trial. Reyanning granules are composed of four botanical drug materials: *Taraxacum* sect. *Taraxacum* [Asteraceae], *Reynoutria japonica* Houtt. [Polygonaceae], *Sonchus brachyotus* DC. [Asteraceae], and *Scutellaria barbata* D.Don [Lamiaceae]. The study batch complied with the National Medical Products Administration standard Z43021027. The scientific names of all source plant materials were verified against the MPNS database. According to published fingerprint analysis, the main marker metabolites of Reyanning granules include chlorogenic acid, caffeic acid, polydatin, luteolin, and emodin (Su et al., 2021). The quality-control testing and chemical fingerprint-based identification of Reyanning Granules were consistent with the reference standards of the *Chinese Pharmacopoeia* (2020 edition).

### 2.5 Intervention

Participants in the experimental group received Bairui granules (batch number: S220401; 5 g per dose, three times daily) plus placebo matching Reyanning granules (batch number: Y220402; 4 g per dose, three times daily). Participants in the control group received Reyanning granules (batch number: 2107502; 4 g per dose, three times daily) plus placebo matching Bairui Granules (batch number: Y220401; 5 g per dose, three times daily). The dose of Bairui Granules was selected according to the approved product instructions and previous adult clinical use reported by Zheng et al. (2020). The placebos were designed to be indistinguishable from the corresponding active preparations in terms of appearance, packaging, labelling, and markings, and to closely match their color, odor, taste, shape, and texture.

### 2.6 Outcome measures

The primary endpoint was the cough-resolution rate after 7 days of treatment. Cough severity was recorded by participants approximately every 12 h using a 4-point ordinal cough symptom scale ranging from 0 (no cough) to 3 (severe cough), based on the cough symptom scoring approach recommended in the *Guideline for the Diagnosis and Treatment of Cough (2015)* issued by the Asthma Group of the Chinese Thoracic Society, Chinese Medical Association.

The secondary endpoints included: (1) time to disappearance of cough and sputum, recorded by participants approximately every 12 h using a symptom diary; (2) the area under the curve (AUC) for cough and sputum severity over time; (3) the change in total Bronchitis Severity Score (BSS) from baseline to day 9 of treatment. The BSS comprises five clinical items—cough, sputum production, rales, chest pain during coughing, and dyspnea—each rated on a scale from 0 (no symptom) to 4 (very severe), yielding a total score ranging from 0 to 20, with higher scores indicating greater disease severity; and (4) the TCM symptom score, which includes daytime cough, nocturnal cough, and sputum symptoms (each scored as 0, 3, 6, or 9 points), as well as fever, aversion to wind, nasal congestion, thirst, and sore throat (each scored from 0 to 3 points). The composite TCM symptom score ranges from 0 (no symptoms) to 42 (maximum severity). Cough resolution for the primary endpoint was operationally defined as disappearance of both daytime and nocturnal cough, with cough scores of 0 for both assessments sustained for at least 24 h. The Day-7 cough-resolution rate was calculated as the proportion of participants who met this cough-resolution criterion by the end of the 7-day treatment period. Sputum disappearance was defined as a sputum score of 0 sustained for at least 24 h. The time elapsed between the first dose of the study medication and the first occurrence of the criteria was recorded as the time taken for cough and sputum to disappear, respectively. Detailed scoring criteria are provided in Supplementary Tables S2–5. All clinical outcome assessments, including the investigator-rated BSS and review of participant symptom diaries, were performed or verified by trained outcome assessors who were blinded to treatment allocation.

Safety was evaluated throughout the study period. Safety outcomes included adverse events related to vital signs, hematological parameters, urinary system, liver function, kidney function, and circulatory system. All adverse events that occurred during the trial were recorded and graded according to the Common Terminology Criteria for Adverse Events (CTCAE), version 5.0.

### 2.7 Sample size

Based on the study by Yin et al. (2009), we assumed that the cough resolution rate on day 7 of treatment would be 50% in the Bairui granules (experimental) group and 27% in the Reyanning granules (control) group. With a two-sided significance level (α) of 0.05, a statistical power of 80%, and an allocation ratio of 2:1 (experimental: control), the required sample size was calculated using PASS 15 software (NCSS Corp.). The calculation indicated that 143 participants were needed in total. Allowing for an anticipated dropout rate of approximately 10%, the planned total sample size was increased to 162 participants, comprising 108 in the experimental group and 54 in the control group.

### 2.8 Statistical analysis

All effectiveness analyses were performed in the full analysis set (FAS), which followed the intention-to-treat (ITT) principle and included all randomized participants who received at least one dose of study medication; safety analyses were conducted in the safety set (SS), comprising all participants who received at least one dose of study medication, and the primary inference for this trial was based on the FAS. Last Observation Carried Forward (LOCF) was prespecified for handling missing diary-derived cough-resolution data in the FAS analysis in order to preserve the ITT. Baseline was defined as the last nonmissing assessment obtained before the first administration of study medication, and baseline characteristics were summarized descriptively (continuous variables as means ± SD or medians with interquartile ranges (IQR), as appropriate, and categorical variables as counts and percentages).

The primary endpoint, the rate of cough resolution after 7 days of treatment, was compared between groups using CMH chi-square test considering central factors. The time to disappearance of cough and sputum was analyzed using the Kaplan-Meier method and compared using the log-rank test. The other secondary endpoints including the AUC for cough and sputum severity over time, the change in total BSS and the TCM symptom score (daytime cough, nocturnal cough, sputum symptoms, and fever, aversion to wind, nasal congestion, thirst, and sore throat), were compared between groups with a two-sample t-test or Mann–Whitney U test, as appropriate. All tests were two-sided, and a *P* value of less than 0.05 was considered to indicate statistical significance. No subgroup analyses were prespecified in the protocol or statistical analysis plan, and no subgroup analyses were conducted. Data were entered into Microsoft Excel 2019 to establish the study database and analyzed with SAS software, version 9.4, with AUC calculations and plots generated in GraphPad Prism, version 10.1.2. Adverse events were coded using MedDRA version 25.1 and graded according to CTCAE version 5.0. Harms were summarized by treatment group as the number of events, number of patients with at least one event, incidence, severity grade, seriousness, relatedness to study medication, adverse events leading to withdrawal, and system organ class/preferred term.

## 3 Results

**FIGURE 1:**
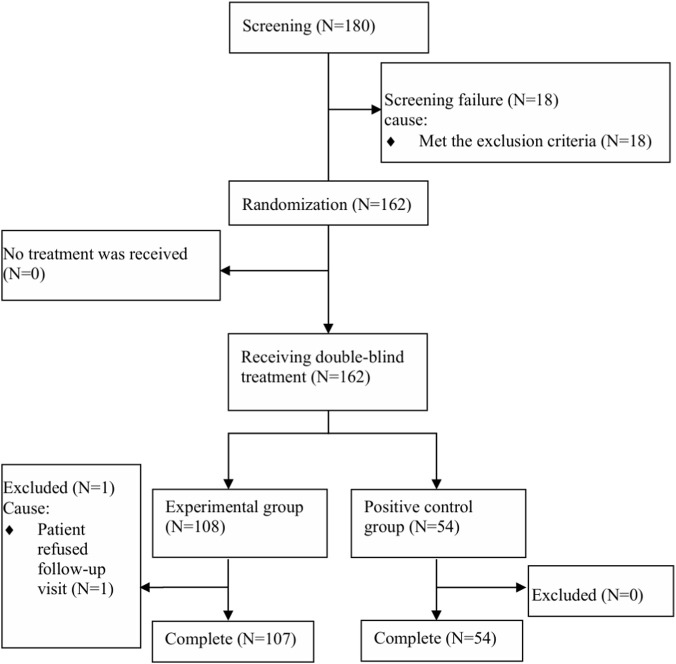
Flow chart of participant distribution (FAS). FAS, full analysis set.

From 30 June 2022, to 12 November 2022, 162 of 180 screened participants were enrolled (108 in the experimental group and 54 in the control group). One participant in the experimental group discontinued the study, resulting in an overall dropout rate of 0.62% (experimental group: 0.93%) (Figure 1). All enrolled participants were included in FAS and SS.

### 3.1 Baseline characteristics

Baseline characteristics were comparable between groups (Table 1). Demographic variables (age, sex, ethnicity, height, and weight) were balanced, with no significant between-group differences (*P* > 0.05). Baseline body temperature, chest X-ray findings, and total TCM symptom scores were also similar between groups (*P* > 0.05).

**TABLE 1:** Comparison of baseline characteristics between the study groups (FAS).

Variables	Experimental group (N = 108)	Control group (N = 54)	P-value
Age (year)^a^	41.62 ± 15.63	42.63 ± 13.89	0.6885
Males^b^	35 (32.41)	17 (31.48)	0.9053
Minority^b^	1 (0.93)	1 (1.85)	1.0000
Height^a^	164.46 ± 8.50	164.57 ± 8.30	0.9371
Weight^a^	63.48 ± 10.67	62.61 ± 11.26	0.6315
Course of disease (hours)^a^	36.27 ± 15.64	34.78 ± 14.34	0.5599
Temperature (°C)^a^	36.52 ± 0.21	36.49 ± 0.29	0.4014
Current comorbidities^b^	34 (31.48)	24 (44.44)	0.1047
Treatment of the disease during the course of the current episode^b^	0 (0.00)	0 (0.00)	1.0000
Chest X-ray examination: Clinically significant abnormalities^b^	9 (8.33)	8 (14.81)	0.2975
Total TCM symptom score^a^	19.01 ± 3.86	19.07 ± 3.46	0.9171

^a^Number, mean, standard deviation; ^b^Number, percentage; TCM, traditional Chinese medicine; FAS, full analysis set.

### 3.2 Primary endpoint

**FIGURE 2:**
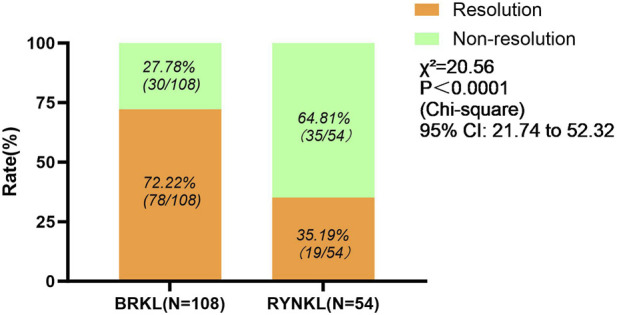
Cough resolution rate after 7 days of treatment (FAS). BRKL, Bairui granules group; RYNKL, Reyanning granules group. CI, confidence Interval. FAS, full analysis set.

After 7 days of treatment, the cough resolution rate was higher in the experimental group than in the control group (72.22% vs. 35.19%; *P* < 0.0001; Figure 2).

### 3.3 Secondary endpoints

#### 3.3.1 Time to disappearance of cough and sputum

**FIGURE 3:**
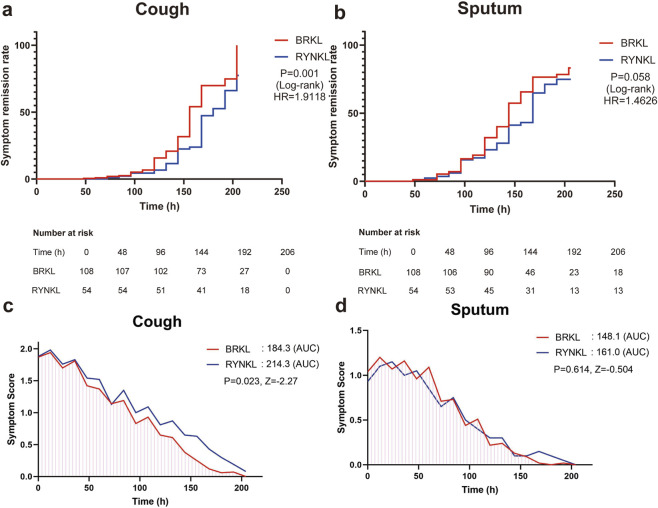
Cough and Sputum: Time to Resolution and Severity AUC Over Time (FAS). **(a)** Time to disappearance of cough; **(b)** Area under the cough severity-time curve; **(c)** Time to disappearance of sputum; **(d)** Area under the sputum severity-time scurve. AUC, the area under the curve. BRKL, Bairui granules group; RYNKL, Reyanning granules group. CI, confidence Interval. HR, hazard Ratio. FAS, full analysis set.

The median time to disappearance of cough was shorter in the experimental group than in the control group (144 h [IQR, 120–156] vs. 168 h [IQR, 138–168]; *P* = 0.001; HR, 1.9118; 95% CI, 1.3049 to 2.8009; Figure 3A). The median time to disappearance of sputum did not differ between groups (120 h [IQR, 96–144] vs. 132 h [IQR, 96–168]; *P* = 0.058; HR, 1.4626; 95% CI, 0.9876 to 2.1659; Figure 3C).

#### 3.3.2 AUC for cough and sputum severity over time

The AUC for cough severity over time was lower in the experimental group than in the control group (184.3 vs. 214.3 score·h; *P* = 0.023; Figure 3B). The AUC for sputum severity over time did not differ between groups (148.1 vs. 161.0 score·h; *P* = 0.614; Figure 3D).

#### 3.3.3 Bronchitis severity score (BSS)

**FIGURE 4:**
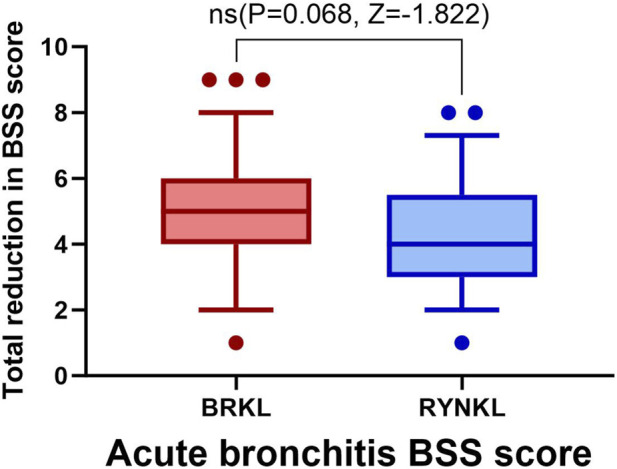
Total BSS on the ninth day of treatment (FAS). BSS, bronchitis severity score. BRKL, Bairui granules group; RYNKL, Reyanning granules group. FAS, full analysis set.

From baseline to day 9, the total BSS decreased by −4.82 ± 3.54 in the experimental group and −4.30 ± 0.71 in the control group, with no significant between-group difference (*P* = 0.068; Figure 4).

#### 3.3.4 TCM symptom score

**FIGURE 5:**
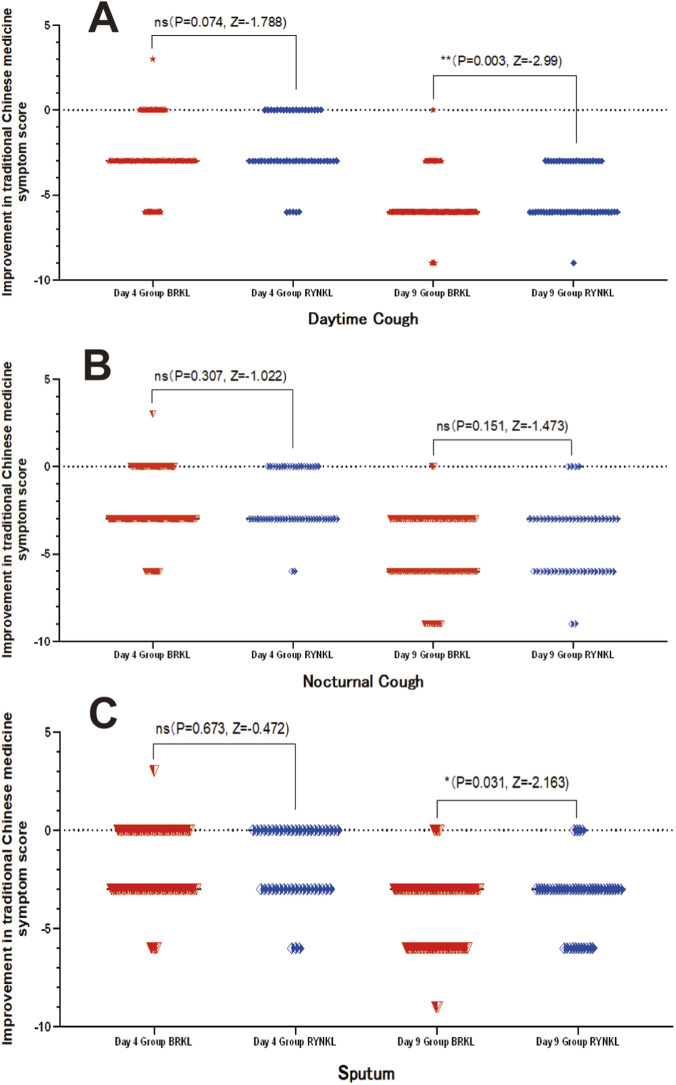
TCM cough and sputum symptom score (FAS). **(A)** Improvement in TCM symptom score for daytime cough; **(B)** Improvement in TCM symptom score for nocturnal cough; **(C)** Improvement in TCM symptom score for sputum. TCM, traditional Chinese medicine. BRKL, Bairui granules group; RYNKL, Reyanning granules group. FAS, full analysis set.

Daytime cough showed no between-group difference on day 4, whereas improvement was greater in the experimental group by day 9 (*P* = 0.003; Figure 5A). No between-group differences were observed for nocturnal cough on either day 4 or day 9 (Figure 5B). For sputum symptoms, groups were comparable on day 4, but improvement was greater in the experimental group on day 9 (*P* = 0.031; Figure 5C). Among other TCM symptoms, between-group differences were observed for aversion to wind on day 4 and sore throat on day 9 (*P* < 0.05), while no differences were detected for aversion to wind on day 9, sore throat on day 4, or thirst and nasal congestion on day 4 or day 9 (Table 2; Supplementary Table S6).

**TABLE 2:** Improvement in TCM single symptom scores (secondary symptoms).

Symptom	Time	Experimental group		Control group		P-value
Aversion to wind	Day4	N = 57	1 (1,1)	N = 30	1 (1,1)	z = −2.31, *P* = 0.021
	Day9	N = 57	1 (1,1)	N = 31	−1 (1,1)	z = −0.651, *P* = 0.515
	​	​		​		(Mann–Whitney U test)
Thirst	Day4	N = 104	1 (1,1)	N = 52	1 (1,1)	z = −0.117, *P* = 0.907
	Day9	N = 104	2 (1,2)	N = 53	1 (1,2)	z = −1.621, *P* = 0.105
	​	​		​		(Mann–Whitney U test)
Sore throat	Day4	N = 99	1 (0,1)	N = 50	1 (0,1)	z = −1.58, *P* = 0.114
	Day9	N = 99	1 (1,1)	N = 51	1 (1,1)	z = −2.11, *P* = 0.035
	​	​		​		(Mann–Whitney U test)
Nasal congestion	Day4	N = 55	1 (1,1)	N = 27	1 (0.5,1)	z = −0.553, *P* = 0.58
	Day9	N = 55	1 (0.5,1)	N = 27	0 (0,1)	z = −1.674, *P* = 0.094
	​	​		​		(Mann–Whitney U test)

Values are presented as median (IQR) of changes from baseline. For each symptom, N refers to the number of participants who had the corresponding symptom at baseline and had non-missing symptom-score data at the specified visit.

TCM, traditional Chinese medicine.

### 3.4 Safety evaluation

Safety analyses were performed in the safety set, which included all randomized participants who received at least one dose of study medication. Adverse events were coded using MedDRA version 25.1 and graded according to CTCAE version 5.0. Harms were summarized by treatment group as the number of events, number of patients with at least one event, incidence, severity grade, seriousness, relatedness to study medication, adverse events leading to withdrawal, and system organ class/preferred term. A total of 20 adverse events (AEs) were reported in 16 patients, corresponding to an overall incidence rate of 9.88%. In the experimental group, 10 patients experienced 13 AEs (incidence rate: 9.26%), whereas in the control group, 6 patients experienced 7 AEs (incidence rate: 11.11%). The treatment-related AEs (definitely, probably, or possibly related to the study intervention) occurred in two patients (three events), all in the experimental group (overall incidence rate: 1.23%; experimental group: 1.85%). No serious adverse events, treatment-related serious adverse events, adverse events leading to withdrawal, or unexpected serious adverse reactions were observed in either group (Tables 3, 4).

**TABLE 3:** All adverse events (SS).

​	Experimental group (N = 108)			Control group (N = 54)			Total (N = 162)		
Project	Number of events	Number of patients	Incidence (%)	Number of events	Number of patients	Incidence (%)	Number of events	Number of patients	Incidence (%)
All adverse events	13	10	9.26	7	6	11.11	20	16	9.88
Related adverse events	3	2	1.85	0	0	0.00	3	2	1.23
Serious adverse events	0	0	0.00	0	0	0.00	0	0	0.00
Serious-related adverse events	0	0	0.00	0	0	0.00	0	0	0.00
Adverse events leading to withdrawal	0	0	0.00	0	0	0.00	0	0	0.00
Associated adverse events leading to withdrawal	0	0	0.00	0	0	0.00	0	0	0.00
Suspicious or unexpected serious adverse reactions	0	0	0.00	0	0	0.00	0	0	0.00

Related adverse events include those definitely, likely, or possibly related to the study drug.

The code dictionary is MedDRA, 25.1 (Chinese version). SS, safety set.

**TABLE 4:** Frequency analysis of all adverse events (SS).

System organ class/Preferred term	Experimental group (N = 108)			Control group (N = 54)			Total (N = 162)		
	Number of events	Number of patients	Incidence (%)	Number of events	Number of patients	Incidence (%)	Number of events	Number of patients	Incidence (%)
Total	13	10	9.26	7	6	11.11	20	16	9.88
All kinds of assessment	5	4	3.70	3	2	3.70	8	6	3.70
Urinary leukocytes were positive	1	1	0.93	1	1	1.85	2	2	1.23
Elevated γ -glutamyltransferase levels	1	1	0.93	0	0	0.00	1	1	0.62
Elevated alanine aminotransferase levels	1	1	0.93	0	0	0.00	1	1	0.62
Urinary red blood cells were positive	0	0	0.00	1	1	1.85	1	1	0.62
Elevated aspartate aminotransferase levels	0	0	0.00	1	1	1.85	1	1	0.62
Elevated bilirubin levels	1	1	0.93	0	0	0.00	1	1	0.62
Elevated blood uric acid levels	1	1	0.93	0	0	0.00	1	1	0.62
Infection and infestation diseases	3	3	2.78	3	3	5.56	6	6	3.70
Urinary tract infection	3	3	2.78	2	2	3.70	5	5	3.09
Gastroenteritis	0	0	0.00	1	1	1.85	1	1	0.62
Various types of neurological diseases	2	2	1.85	0	0	0.00	2	2	1.23
Dizziness	2	2	1.85	0	0	0.00	2	2	1.23
Skin and subcutaneous tissue diseases	2	1	0.93	0	0	0.00	2	1	0.62
Erythra	1	1	0.93	0	0	0.00	1	1	0.62
Pruritus	1	1	0.93	0	0	0.00	1	1	0.62
Hepatobiliary system diseases	0	0	0.00	1	1	1.85	1	1	0.62
Abnormal liver function	0	0	0.00	1	1	1.85	1	1	0.62
Systemic disease and various reactions at the site of administration	1	1	0.93	0	0	0.00	1	1	0.62
Tiredness	1	1	0.93	0	0	0.00	1	1	0.62

The code dictionary is MedDRA25.1 (Chinese version), SS, safety set.

## 4 Discussion

This multicenter, randomized, double-blind, double-dummy, comparator trial showed that Bairui Granules, initiated within 72 h after symptom onset, were associated with better cough-related outcomes than Reyanning Granules in adults with acute bronchitis presenting with wind-heat invading lung syndrome. Specifically, the Bairui group had a higher Day-7 cough-resolution rate and a shorter median time to cough resolution. The AUC for cough severity was also lower in the Bairui group, suggesting a lower cumulative cough burden during follow-up. However, not all secondary outcomes showed statistically significant between-group differences; the time to sputum resolution, sputum-severity AUC, and total BSS improvement did not differ significantly between groups.

Our study was the first trial to evaluate the effectiveness of Bairui granules in treating adult patients with acute bronchitis. It found that participants experienced a higher rate of cough resolution (72.22% vs. 35.19%) after 7 days of treatment and a shorter duration of cough symptoms (144 h vs. 168 h) with a median duration of 6 days when taking Bairui granules. Bairui granules also significantly reduced cough severity and decreased sputum symptoms score. These outcomes were similar to other studies in treating children with acute bronchitis (Sun, 2021; Feng et al., 2020; Xu et al., 2023b). Cough is the most common symptom of acute bronchitis. Acute bronchitis-related cough commonly persists for 2–3 weeks, with pooled estimates of approximately 18 days, and acute cough can impair quality of life by causing discomfort, sleep disturbance, and limitations in daily activities (Ebell et al., 2013; Drewitz et al., 2025). The persistent cough symptom may also lead to the inappropriate use of antibiotics. However, many high-quality evidences have shown that antibiotics do not provide rigorous benefit or improve the cure rate in patients with acute bronchitis (Little et al., 2005; Smith et al., 2017). Therefore, a 1-day earlier resolution of cough may be meaningful for some patients, especially when considered together with the higher Day-7 cough-resolution rate and lower cough-severity AUC observed in the Bairui group. Prior UPLC–MS/MS and phytochemical studies have reported that Bairui Granules contain multiple plant metabolites, including alkaloids, flavonoids, organic acids, glycosides, terpenes, and lactones, with flavonoids—particularly kaempferol—constituting the putative bioactive metabolites (Li et al., 2021; Zhang et al., 2024; Peng et al., 2024). Experimental studies have suggested that kaempferol has antitussive and anti-infective effects; D-mannitol and succinic acid exhibit anti-asthmatic benefits; and flavonoids inhibit both Gram-positive and Gram-negative bacteria (Zhang et al., 2024; Peng et al., 2024). These findings provide a biologically plausible hypothesis for the cough and sputum improvements observed in the present trial.

Although the primary endpoint showed a statistically significant between-group difference, the change in total BSS did not reach statistical significance. This discrepancy may be explained by differences in the clinical dimensions captured by these endpoints. The primary endpoint was a cough-specific outcome derived from participant symptom diaries and required disappearance of both daytime and nocturnal cough sustained for at least 24 h. In contrast, the BSS is a composite measure that includes cough, sputum production, rales, chest pain during coughing, and dyspnea. In addition, this study was conducted as a post-marketing indication-expansion trial. The 2:1 randomization ratio was chosen to increase the number of participants exposed to Bairui Granules and to provide more safety data for the investigational treatment. However, compared with a 1:1 allocation at the same total sample size, this allocation ratio may have reduced statistical efficiency and precision for between-group comparisons, particularly for secondary endpoints.

In terms of TCM symptoms, we also evaluated the single symptom scores of aversion to wind, thirst, sore throat, and nasal congestion. These symptoms, in TCM theory, are the characteristics of acute bronchitis presented with “wind-heat invading the lung” syndrome (Wang et al., 2013). The results showed that compared to Reyanning granules, Bairui granules can quickly alleviate the symptoms of aversion to wind, and significantly reduce the symptoms of sore throat. For patients with respiratory tract infections, the rapid relief of aversion to wind symptom can significantly improve activity tolerance, restore normal temperature regulation function, and thus greatly improve the overall comfort and rehabilitation quality of patients. In addition, shortening sore throat duration can directly improve swallowing function, promote nutrient intake, restore speech ability, and comprehensively improve the quality of life of patients during the disease (Kitano and Tsuzuki, 2025).

Bairui granules were generally well tolerated in this trial. The overall incidence of adverse events was 9.88%, with rates of 9.26% in the Bairui group and 11.11% in the control group. All adverse events were mild to moderate according to CTCAE v5.0, including grade 1–2 events in the Bairui group and grade 1 events in the control group, and most events were considered unrelated to treatment. Together with previous clinical reports in respiratory diseases, these findings suggest that short-term use of Bairui granules is generally well tolerated in the studied population.

Our trial has some limitations. First, this study was designed as a double-blind, double-dummy, active-comparator trial and did not include an inactive placebo-only arm. Acute bronchitis is usually self-limiting; therefore, this design cannot determine the absolute efficacy of Bairui Granules beyond natural recovery. Future randomized trials including inactive placebo arm is needed to quantify the absolute treatment effect and to determine whether Bairui Granules provide benefit beyond spontaneous symptom resolution. Second, the primary endpoint was symptom-based and patient-reported. Although the cough diary was based on a cough symptom scoring approach recommended by the Chinese Thoracic Society guideline and allowed frequent assessment of cough resolution, it remained subjective. Future studies should incorporate objective cough-frequency monitoring, inflammatory biomarkers, pulmonary-function tests. Third, all participants were required to meet the TCM diagnostic criteria for wind-heat invading lung syndrome, and the study excluded pregnant or breastfeeding individuals, participants who had used antibiotics within 48 h before enrollment, and patients with severe diseases involving major organs or systems. Therefore, the findings may not be applicable to broader acute bronchitis populations, including patients with other TCM syndrome patterns or higher-risk populations excluded from this trial. Future studies should evaluate the safety and effectiveness of Bairui Granules in these populations. Finally, the sample-size calculation was based on limited older evidence from Yin et al. (2009), and future trials should use more contemporary pilot data to inform effect-size assumptions.

## 5 Conclusion

Bairui granules resulted in a higher cough-resolution rate and faster cough resolution than Reyanning granules in adults with acute bronchitis. The treatment was well tolerated.

## Data Availability

The raw data supporting the conclusions of this article will be made available by the authors, without undue reservation.
