# Supplementary material for: Bairui granules versus Reyanning granules in adults with acute bronchitis: a multicenter, randomized, double-blind, double-dummy, comparative trial
Source: Front Pharmacol. 2026 Jul 16;17:1860478. doi: 10.3389/fphar.2026.1860478 (PMC13422438; doi:10.3389/fphar.2026.1860478)
Supplement: Supplementary file 1 [file Supplementaryfile1.docx]

Bairui granules versus Reyanning granules in adults with acute bronchitis: a multicenter, randomized, double-blind, double-dummy, comparative trial

**Youqiang Wu^1†^, Guantong Shen^2†^_,_ Shuyang Ji^1^, Guoxing Liu^1^, Weicheng Nie^2^, Bei Xue^1^, Chen Zuo^1^, Yingjie Du^1^,** **Jingyi Qi^3^, Mingzhe Wang^1^*, Chengjun Ban^1^*, Miao Cheng^1^***

^1^ Respiratory Department, Dongzhimen Hospital, Beijing University of Chinese Medicine, Beijing, China

^2^ Respiratory Department, Beijing University of Chinese Medicine Third Affiliated Hospital, Beijing, China

^3^ OISE Department of Applied Psychology & Human Development, Univeristy of Toronto, Toronto, Canada

***Correspondence:**Mingzhe Wang*(wmz0723@foxmail.com)

Chengjun Ban* (13810654835@139.com)

Miao Cheng* ([chengxinxin321@126.com](mailto:chengxinxin321@126.com))

**†** These authors have contributed equal to this work


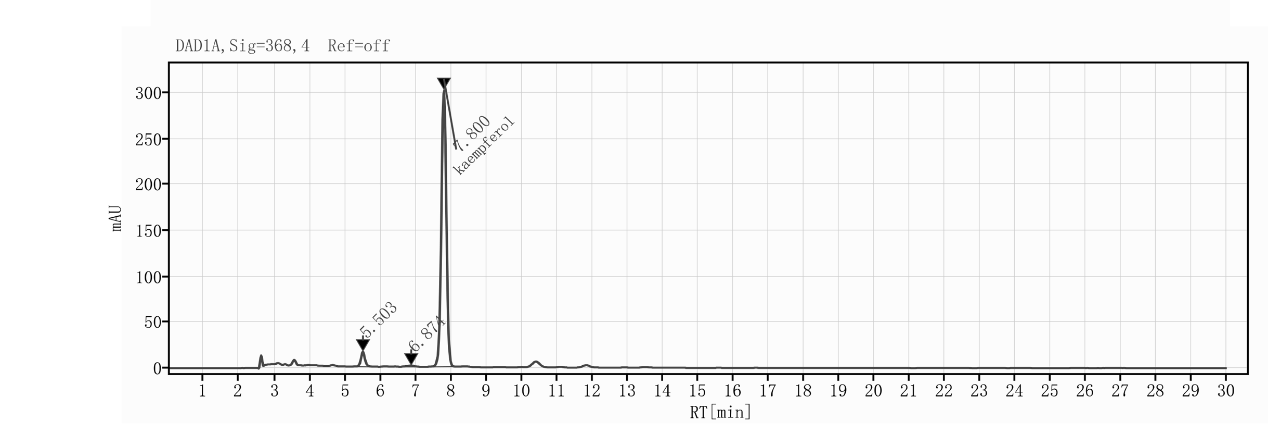


**Supplementary Figure S1.** Determination of the content of Bairui granules (Kaempferol).

**Supplementary Table S1** Clinical trial sites and investigator

| Site | Investigators |
| --- | --- |
| Dongzhimen Hospital Affiliated to Beijing University of Chinese Medicine (BUCM), Beijing, China | Chengjun Ban |
| Luohe hospital of Traditional Chinese Medicine | Qiang Li |
| The Third People's Hospital of Luoyang City | Min Zhou |
| Kaifeng hospital of Traditional Chinese Medicine | Qixiang Wu |
| The Second Hospital of Shandong University | Jun Wang |
| Second Affiliated Hospital of Heilongjiang University of Traditional Chinese Medicine | Shanjun Yang |
| Chengdu University Affiliated Hospital | Hui Zhou |

**Supplementary** **Table S2** Bronchiolitis severity score (BSS)

| Symptom | Value | | | | |
| --- | --- | --- | --- | --- | --- |
|  | 0 | 1 | 2 | 3 | 4 |
| Cough | No symptom | Mild | Moderate | Serious | Very serious |
| Sputum | No symptom | Mild | Moderate | Serious | Very serious |
| Rale | No symptom | Mild | Moderate | Serious | Very serious |
| Chest pain caused by cough | No symptom | Mild | Moderate | Serious | Very serious |
| Dyspnea | No symptom | Mild | Moderate | serious | Very serious |

**Supplementary Table S3** Cough symptom scale

| Symptom | Value | | | |
| --- | --- | --- | --- | --- |
|  | 0 | 1 | 2 | 3 |
| Daytime cough | No cough during the day | Occasional brief cough | Frequent coughing that mildly affects daily activities | Frequent coughing that seriously affects daily activities |
| Nocturnal cough | No cough during the night | Brief or occasional cough when falling asleep | Mild disturbance of sleep at night due to cough | Serious disturbance of sleep at night due to cough |

**Supplementary Table S4** Sputum symptom scale

| Symptom | Value | | | |
| --- | --- | --- | --- | --- |
|  | 0 | 1 | 2 | 3 |
| Cough and phlegm | Normal | Occasional cough with a small amount of sputum and saliva | Cough with sputum, with mild wheezing due to retention of phlegm in the throat | Cough with a large amount of sputum, with wheezing due to retention of phlegm in the throat |

**Supplementary Table S5** Traditional Chinese medicine symptom scale

| Symptom | Value | | | |
| --- | --- | --- | --- | --- |
| Primary symptoms | 0 Points | 3 Points | 6 Points | 9 Points |
| Cough  (daytime) | No cough | Occasional brief cough | Frequent cough that mildly affects  daily activities | Frequent cough that seriously affects daily activities |
| Cough  (nocturnal) | No cough | Brief or occasional cough at night during sleep | Cough that slightly affects nocturnal sleep | Cough that severely affects nocturnal sleep |
| Sputum | No sputum | Small amount, white, slightly sticky phlegm that is easy to cough out | Moderate amount, white-yellow sticky phlegm that is relatively easy to cough out | Large amount, thick, yellow, sticky phlegm that is difficult to cough out |
| Secondary symptoms | 0 Points | 1 Points | 2 Points | 3 Points |
| Fever | The maximum axillary temperature at 24 h before diagnosis was 37.2℃ | The maximum axillary temperature at 24 h before diagnosis was 37.3–37.9℃ | The maximum axillary temperature at 24 h before diagnosis was 38.0–38.4℃ | The highest axillary temperature was > 38.5℃ at 24 h before diagnosis |
| Aversion to wind | No aversion to wind | Mild sensitivity to cold | Obviously sensitive to cold, requiring additional clothing and a quilt | Sensitive to cold, additional clothing does not provide relief |
| Rhinobyon | No rhinobyon | Minor nasal congestion that does not affect breathing | Nasal congestion, breathing nasal ringing | Nose obstruction, open mouth breathing |
| Thirst | No thirst | No need to drink water despite feeling thirsty | Drinking water in response to thirst | Drinking water frequently when feeling thirsty |
| Sore throat | No sore throat | Dry or slightly sore throat | Moderate sore throat that is noticeable during swallowing | Pain in the pharynx, making swallowing difficult |
