# Supplementary material for: Bairui granules versus Reyanning granules in adults with acute bronchitis: a multicenter, randomized, double-blind, double-dummy, comparative trial
Source: Front Pharmacol. 2026 Jul 16;17:1860478. doi: 10.3389/fphar.2026.1860478 (PMC13422438; doi:10.3389/fphar.2026.1860478)
Supplement: Supplementary file 2 [file Table1.docx]

| Site | Investigators |
| --- | --- |
| Dongzhimen Hospital Affiliated to Beijing University of Chinese Medicine (BUCM), Beijing, China | Chengjun Ban |
| Luohe hospital of Traditional Chinese Medicine | Qiang Li |
| The Third People's Hospital of Luoyang City | Min Zhou |
| Kaifeng hospital of Traditional Chinese Medicine | Qixiang Wu |
| The Second Hospital of Shandong University | Jun Wang |
| Second Affiliated Hospital of Heilongjiang University of Traditional Chinese Medicine | Shanjun Yang |
| Chengdu University Affiliated Hospital | Hui Zhou |

**Supplementary Table S1** Clinical trial sites and investigator

**Supplementary** **Table S2** Bronchiolitis severity score (BSS)

| Symptom | Value | | | | |
| --- | --- | --- | --- | --- | --- |
|  | 0 | 1 | 2 | 3 | 4 |
| Cough | No symptom | Mild | Moderate | Serious | Very serious |
| Sputum | No symptom | Mild | Moderate | Serious | Very serious |
| Rale | No symptom | Mild | Moderate | Serious | Very serious |
| Chest pain caused by cough | No symptom | Mild | Moderate | Serious | Very serious |
| Dyspnea | No symptom | Mild | Moderate | serious | Very serious |

**Supplementary Table S3** Cough symptom scale

| Symptom | Value | | | |
| --- | --- | --- | --- | --- |
|  | 0 | 1 | 2 | 3 |
| Daytime cough | No cough during the day | Occasional brief cough | Frequent coughing that mildly affects daily activities | Frequent coughing that seriously affects daily activities |
| Nocturnal cough | No cough during the night | Brief or occasional cough when falling asleep | Mild disturbance of sleep at night due to cough | Serious disturbance of sleep at night due to cough |

**Supplementary Table S4** Sputum symptom scale

| Symptom | Value | | | |
| --- | --- | --- | --- | --- |
|  | 0 | 1 | 2 | 3 |
| Cough and phlegm | Normal | Occasional cough with a small amount of sputum and saliva | Cough with sputum, with mild wheezing due to retention of phlegm in the throat | Cough with a large amount of sputum, with wheezing due to retention of phlegm in the throat |

**Supplementary Table S5** Traditional Chinese medicine symptom scale

| Symptom | Value | | | |
| --- | --- | --- | --- | --- |
| Primary symptoms | 0 Points | 3 Points | 6 Points | 9 Points |
| Cough  (daytime) | No cough | Occasional brief cough | Frequent cough that mildly affects  daily activities | Frequent cough that seriously affects daily activities |
| Cough  (nocturnal) | No cough | Brief or occasional cough at night during sleep | Cough that slightly affects nocturnal sleep | Cough that severely affects nocturnal sleep |
| Sputum | No sputum | Small amount, white, slightly sticky phlegm that is easy to cough out | Moderate amount, white-yellow sticky phlegm that is relatively easy to cough out | Large amount, thick, yellow, sticky phlegm that is difficult to cough out |
| Secondary symptoms | 0 Points | 1 Points | 2 Points | 3 Points |
| Fever | The maximum axillary temperature at 24 h before diagnosis was 37.2℃ | The maximum axillary temperature at 24 h before diagnosis was 37.3–37.9℃ | The maximum axillary temperature at 24 h before diagnosis was 38.0–38.4℃ | The highest axillary temperature was > 38.5℃ at 24 h before diagnosis |
| Aversion to wind | No aversion to wind | Mild sensitivity to cold | Obviously sensitive to cold, requiring additional clothing and a quilt | Sensitive to cold, additional clothing does not provide relief |
| Rhinobyon | No rhinobyon | Minor nasal congestion that does not affect breathing | Nasal congestion, breathing nasal ringing | Nose obstruction, open mouth breathing |
| Thirst | No thirst | No need to drink water despite feeling thirsty | Drinking water in response to thirst | Drinking water frequently when feeling thirsty |
| Sore throat | No sore throat | Dry or slightly sore throat | Moderate sore throat that is noticeable during swallowing | Pain in the pharynx, making swallowing difficult |

**Supplementary Table S6** Distribution of TCM single symptom scores (secondary symptoms).

| **Symptom** | **Time** | **Reduction** | **BRKL** | **RYNKL** |
| --- | --- | --- | --- | --- |
| Aversion to wind | Day4 | 0 | 6 (10.5%) | 7 (23.3%) |
|  |  | 1 | 44 (77.2%) | 23 (76.7%) |
|  |  | 2 | 7 (12.3%) | 0 (0%) |
|  | Day9 | 0 | 0 (0%) | 1 (3.2%） |
|  |  | 1 | 46 (80.7%) | 25 (80.7%) |
|  |  | 2 | 11 (19.3%) | 5 (16.1%) |
| Thirst | Day4 | -1 | 2 (1.9%) | 2 (3.8%) |
|  |  | 0 | 13 (12.5%) | 7 (13.5%) |
|  |  | 1 | 71 (68.3%) | 33 (63.5%) |
|  |  | 2 | 18 (17.3%) | 10 (19.2%) |
|  | Day9 | -1 | 1 (1.0%) | 1 (1.9%) |
|  |  | 0 | 5 (4.8%) | 1 (1.9%) |
|  |  | 1 | 37(35.6%) | 28 (52.8%) |
|  |  | 2 | 57 (54.8%) | 22 (41.5%) |
|  |  | 3 | 4 (3.8%) | 1 (1.9%) |
| Sore throat | Day4 | -1 | 0 (0%) | 1 (2.0%) |
|  |  | 0 | 26 (26.3%) | 16 (32.0%) |
|  |  | 1 | 63 (63.6%) | 32 (64.0%) |
|  |  | 2 | 10 (10.1%) | 13 (2.0%) |
|  | Day9 | 0 | 2 (2.1%) | 6 (11.8%) |
|  |  | 1 | 84 (84.8%) | 41 (80.4%) |
|  |  | 2 | 13 (13.1%) | 4 (7.8%) |
| Rhinobyon | Day4 | -1 | 1 (1.8%) | 2 (7.4%) |
|  |  | 0 | 11 (20.0%) | 5 (18.5%) |
|  |  | 1 | 38 (69.1%) | 18 (66.7%) |
|  |  | 2 | 5 (9.1%) | 2 (7.4%) |
|  | Day9 | 0 | 41 (74.5%) | 18 (66.7%) |
|  |  | 1 | 12 (21.8%) | 7 (25.9%) |
|  |  | 2 | 2 (3.7%) | 2 (7.4%) |
